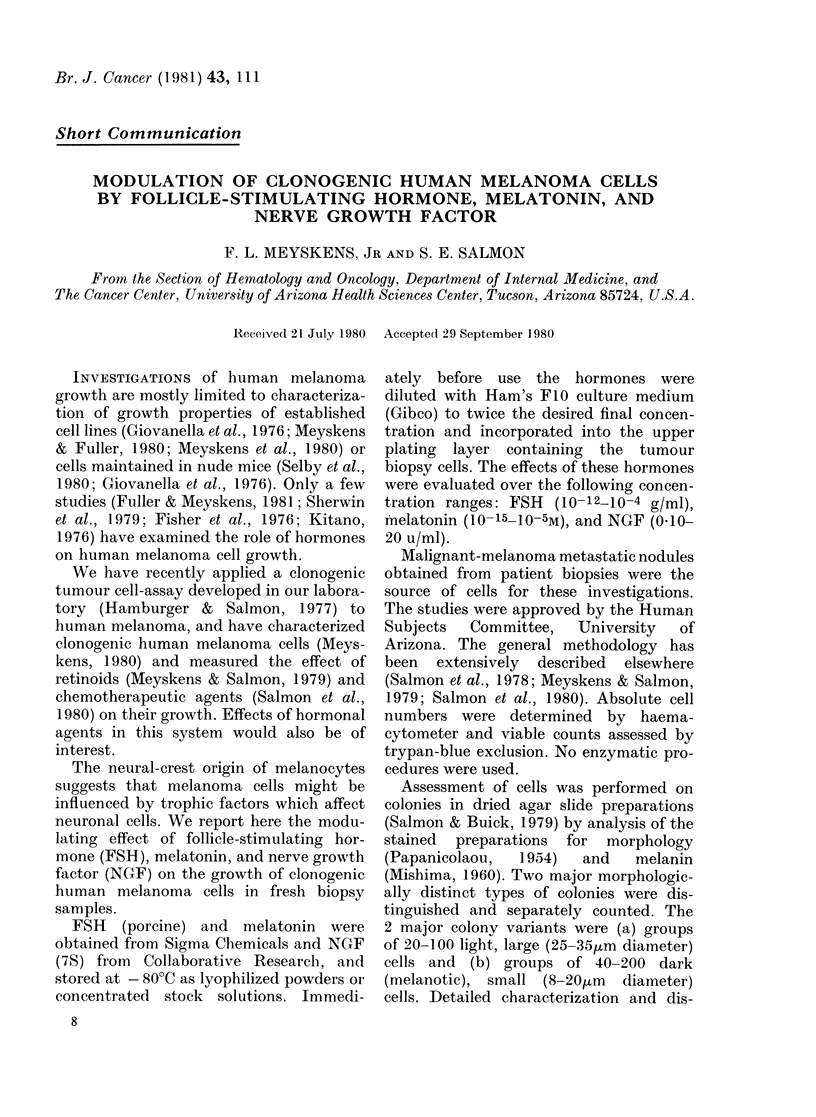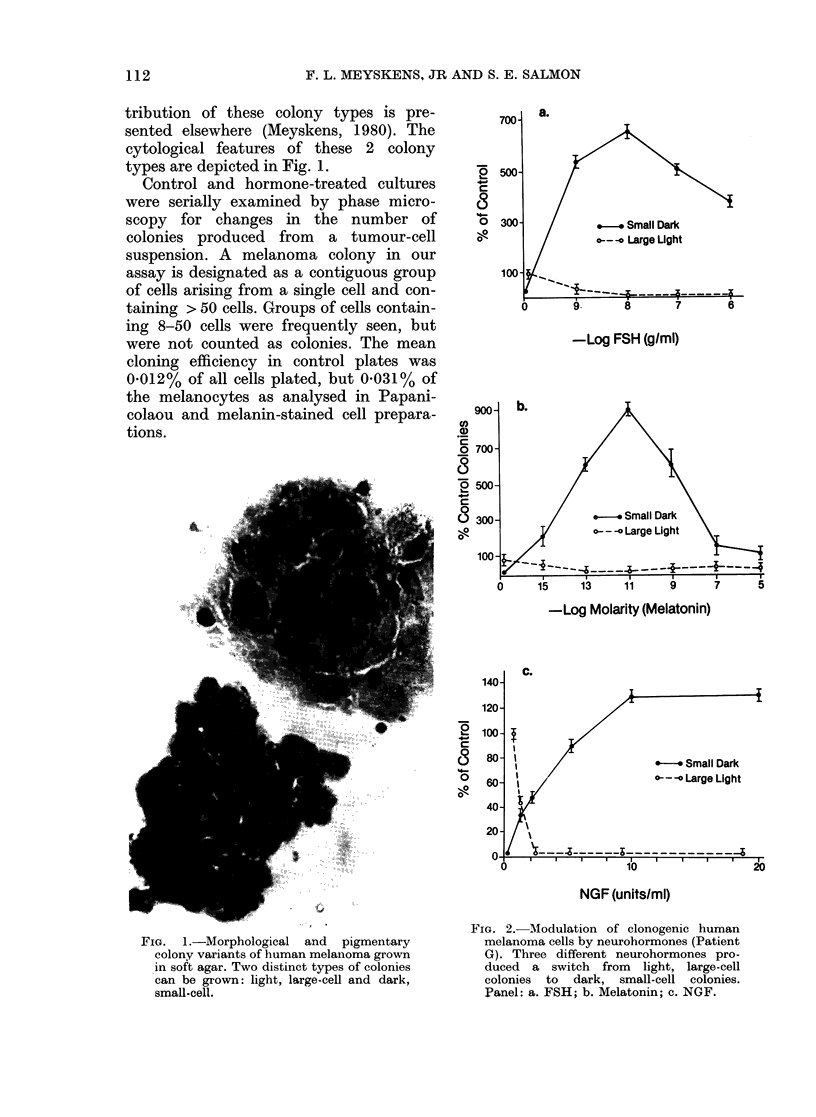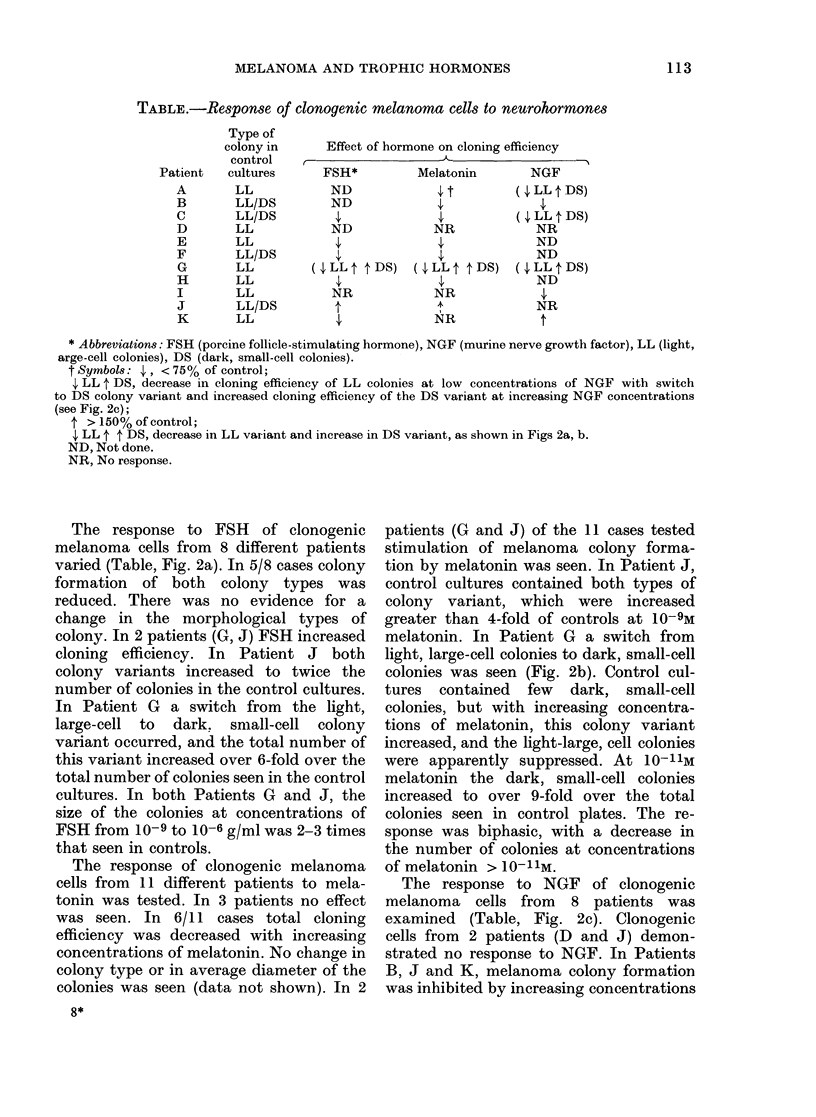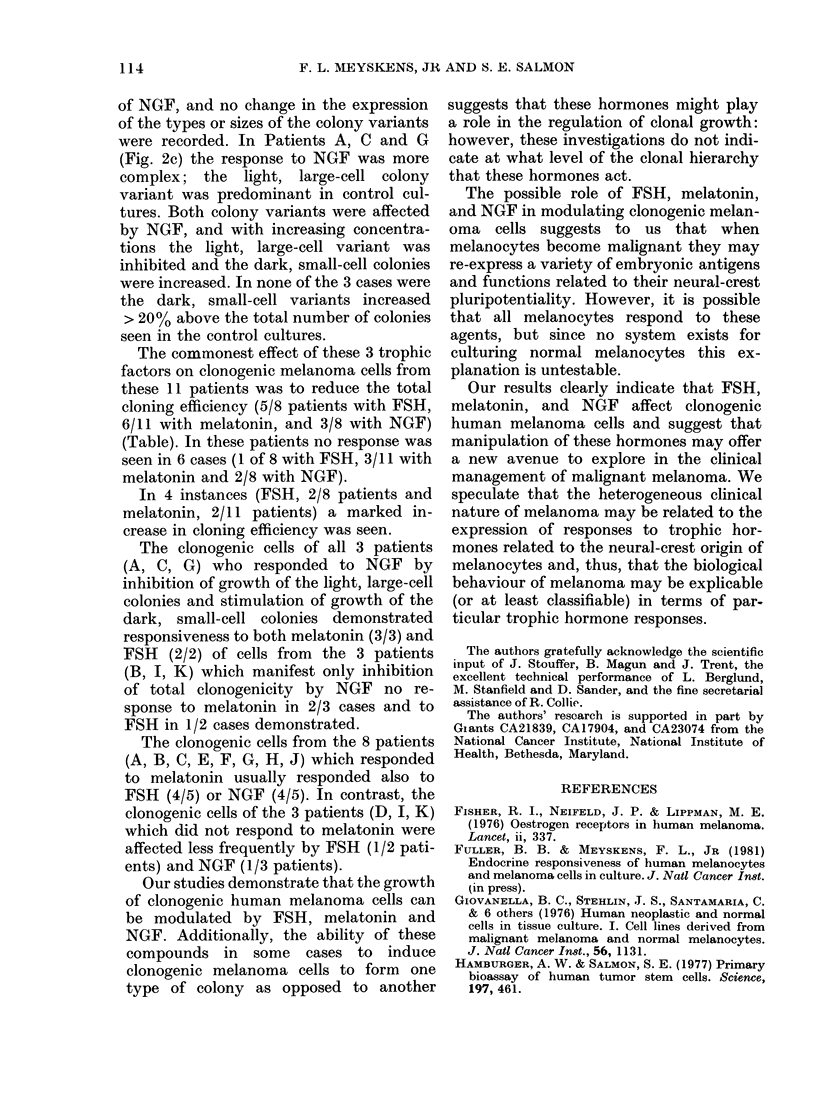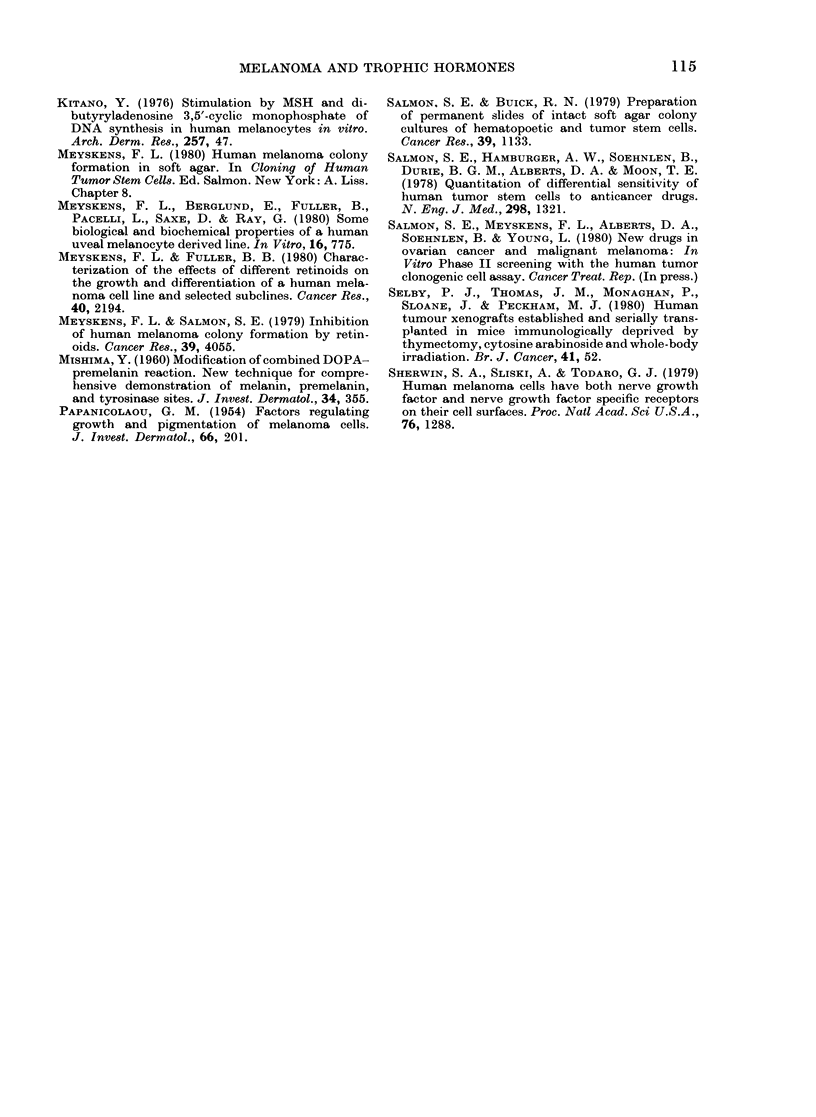# Modulation of clonogenic human melanoma cells by follicle-stimulating hormone, melatonin, and nerve growth factor.

**DOI:** 10.1038/bjc.1981.17

**Published:** 1981-01

**Authors:** F. L. Meyskens, S. E. Salmon

## Abstract

**Images:**


*Br. J. Cancer* (1981) **43**, 111

***Short Communication***

### MODULATION OF CLONOGENIC HUMAN MELANOMA CELLS BY FOLLICLE-STIMULATING HORMONE, MELATONIN, AND NERVE GROWTH FACTOR

F. L. MEYSKENS, JR AND S. E. SALMON

*From the Section of Hematology and Oncology, Department of Internal Medicine, and The Cancer Center, University of Arizona Health Sciences Center, Tucson, Arizona* 85724, *U.S.A.*

Received 21 July 1980 Accepted 29 September 1980

INVESTIGATIONS of human melanoma growth are mostly limited to characterization of growth properties of established cell lines (Giovanella *et al.*, 1976; Meyskens & Fuller, 1980; Meyskens *et al.*, 1980) or cells maintained in nude mice (Selby *et al.*, 1980; Giovanella *et al.*, 1976). Only a few studies (Fuller & Meyskens, 1981; Sherwin *et al.*, 1979; Fisher *et al.*, 1976; Kitano, 1976) have examined the role of hormones on human melanoma cell growth.

We have recently applied a clonogenic tumour cell-assay developed in our laboratory (Hamburger & Salmon, 1977) to human melanoma, and have characterized clonogenic human melanoma cells (Meyskens, 1980) and measured the effect of retinoids (Meyskens & Salmon, 1979) and chemotherapeutic agents (Salmon *et al.*, 1980) on their growth. Effects of hormonal agents in this system would also be of interest.

The neural-crest origin of melanocytes suggests that melanoma cells might be influenced by trophic factors which affect neuronal cells. We report here the modulating effect of follicle-stimulating hormone (FSH), melatonin, and nerve growth factor (NGF) on the growth of clonogenic human melanoma cells in fresh biopsy samples.

FSH (porcine) and melatonin were obtained from Sigma Chemicals and NGF (7S) from Collaborative Research, and stored at $-80°C$ as lyophilized powders or concentrated stock solutions. Immediately before use the hormones were diluted with Ham's F10 culture medium (Gibco) to twice the desired final concentration and incorporated into the upper plating layer containing the tumour biopsy cells. The effects of these hormones were evaluated over the following concentration ranges: FSH ($10^{-12}$–$10^{-4}$ g/ml), melatonin ($10^{-15}$–$10^{-5}$M), and NGF (0·10–20 u/ml).

Malignant-melanoma metastatic nodules obtained from patient biopsies were the source of cells for these investigations. The studies were approved by the Human Subjects Committee, University of Arizona. The general methodology has been extensively described elsewhere (Salmon *et al.*, 1978; Meyskens & Salmon, 1979; Salmon *et al.*, 1980). Absolute cell numbers were determined by haemacytometer and viable counts assessed by trypan-blue exclusion. No enzymatic procedures were used.

Assessment of cells was performed on colonies in dried agar slide preparations (Salmon & Buick, 1979) by analysis of the stained preparations for morphology (Papanicolaou, 1954) and melanin (Mishima, 1960). Two major morphologically distinct types of colonies were distinguished and separately counted. The 2 major colony variants were (a) groups of 20–100 light, large (25–35$\mu$m diameter) cells and (b) groups of 40–200 dark (melanotic), small (8–20$\mu$m diameter) cells. Detailed characterization and distribution of these colony types is presented elsewhere (Meyskens, 1980). The cytological features of these 2 colony types are depicted in Fig. 1.

Control and hormone-treated cultures were serially examined by phase microscopy for changes in the number of colonies produced from a tumour-cell suspension. A melanoma colony in our assay is designated as a contiguous group of cells arising from a single cell and containing > 50 cells. Groups of cells containing 8–50 cells were frequently seen, but were not counted as colonies. The mean cloning efficiency in control plates was 0·012% of all cells plated, but 0·031% of the melanocytes as analysed in Papanicolaou and melanin-stained cell preparations.

FIG. 1.—Morphological and pigmentary colony variants of human melanoma grown in soft agar. Two distinct types of colonies can be grown: light, large-cell and dark, small-cell.

FIG. 2.—Modulation of clonogenic human melanoma cells by neurohormones (Patient G). Three different neurohormones produced a switch from light, large-cell colonies to dark, small-cell colonies. Panel: a. FSH; b. Melatonin; c. NGF.

TABLE.—*Response of clonogenic melanoma cells to neurohormones*

| Patient | Type of colony in control cultures | Effect of hormone on cloning efficiency | | |
|---|---|---|---|---|
| | | FSH* | Melatonin | NGF |
| A | LL | ND | ↓ † | (↓ LL ↑ DS) |
| B | LL/DS | ND | ↓ | ↓ |
| C | LL/DS | ↓ | ↓ | (↓ LL ↑ DS) |
| D | LL | ND | NR | NR |
| E | LL | ↓ | ↓ | ND |
| F | LL/DS | ↓ | ↓ | ND |
| G | LL | (↓ LL ↑ ↑ DS) | (↓ LL ↑ ↑ DS) | (↓ LL ↑ DS) |
| H | LL | ↓ | ↓ | ND |
| I | LL | NR | NR | ↓ |
| J | LL/DS | ↑ | ↑ | NR |
| K | LL | ↓ | NR | ↑ |

* *Abbreviations:* FSH (porcine follicle-stimulating hormone), NGF (murine nerve growth factor), LL (light, arge-cell colonies), DS (dark, small-cell colonies).

† *Symbols:* ↓, < 75% of control;

↓ LL ↑ DS, decrease in cloning efficiency of LL colonies at low concentrations of NGF with switch to DS colony variant and increased cloning efficiency of the DS variant at increasing NGF concentrations (see Fig. 2c);

↑ > 150% of control;

↓ LL ↑ ↑ DS, decrease in LL variant and increase in DS variant, as shown in Figs 2a, b.

ND, Not done.

NR, No response.

The response to FSH of clonogenic melanoma cells from 8 different patients varied (Table, Fig. 2a). In 5/8 cases colony formation of both colony types was reduced. There was no evidence for a change in the morphological types of colony. In 2 patients (G, J) FSH increased cloning efficiency. In Patient J both colony variants increased to twice the number of colonies in the control cultures. In Patient G a switch from the light, large-cell to dark, small-cell colony variant occurred, and the total number of this variant increased over 6-fold over the total number of colonies seen in the control cultures. In both Patients G and J, the size of the colonies at concentrations of FSH from $10^{-9}$ to $10^{-6}$ g/ml was 2–3 times that seen in controls.

The response of clonogenic melanoma cells from 11 different patients to melatonin was tested. In 3 patients no effect was seen. In 6/11 cases total cloning efficiency was decreased with increasing concentrations of melatonin. No change in colony type or in average diameter of the colonies was seen (data not shown). In 2 patients (G and J) of the 11 cases tested stimulation of melanoma colony formation by melatonin was seen. In Patient J, control cultures contained both types of colony variant, which were increased greater than 4-fold of controls at $10^{-9}$M melatonin. In Patient G a switch from light, large-cell colonies to dark, small-cell colonies was seen (Fig. 2b). Control cultures contained few dark, small-cell colonies, but with increasing concentrations of melatonin, this colony variant increased, and the light-large, cell colonies were apparently suppressed. At $10^{-11}$M melatonin the dark, small-cell colonies increased to over 9-fold over the total colonies seen in control plates. The response was biphasic, with a decrease in the number of colonies at concentrations of melatonin $> 10^{-11}$M.

The response to NGF of clonogenic melanoma cells from 8 patients was examined (Table, Fig. 2c). Clonogenic cells from 2 patients (D and J) demonstrated no response to NGF. In Patients B, J and K, melanoma colony formation was inhibited by increasing concentrations of NGF, and no change in the expression of the types or sizes of the colony variants were recorded. In Patients A, C and G (Fig. 2c) the response to NGF was more complex; the light, large-cell colony variant was predominant in control cultures. Both colony variants were affected by NGF, and with increasing concentrations the light, large-cell variant was inhibited and the dark, small-cell colonies were increased. In none of the 3 cases were the dark, small-cell variants increased $> 20\%$ above the total number of colonies seen in the control cultures.

The commonest effect of these 3 trophic factors on clonogenic melanoma cells from these 11 patients was to reduce the total cloning efficiency (5/8 patients with FSH, 6/11 with melatonin, and 3/8 with NGF) (Table). In these patients no response was seen in 6 cases (1 of 8 with FSH, 3/11 with melatonin and 2/8 with NGF).

In 4 instances (FSH, 2/8 patients and melatonin, 2/11 patients) a marked increase in cloning efficiency was seen.

The clonogenic cells of all 3 patients (A, C, G) who responded to NGF by inhibition of growth of the light, large-cell colonies and stimulation of growth of the dark, small-cell colonies demonstrated responsiveness to both melatonin (3/3) and FSH (2/2) of cells from the 3 patients (B, I, K) which manifest only inhibition of total clonogenicity by NGF no response to melatonin in 2/3 cases and to FSH in 1/2 cases demonstrated.

The clonogenic cells from the 8 patients (A, B, C, E, F, G, H, J) which responded to melatonin usually responded also to FSH (4/5) or NGF (4/5). In contrast, the clonogenic cells of the 3 patients (D, I, K) which did not respond to melatonin were affected less frequently by FSH (1/2 patients) and NGF (1/3 patients).

Our studies demonstrate that the growth of clonogenic human melanoma cells can be modulated by FSH, melatonin and NGF. Additionally, the ability of these compounds in some cases to induce clonogenic melanoma cells to form one type of colony as opposed to another suggests that these hormones might play a role in the regulation of clonal growth: however, these investigations do not indicate at what level of the clonal hierarchy that these hormones act.

The possible role of FSH, melatonin, and NGF in modulating clonogenic melanoma cells suggests to us that when melanocytes become malignant they may re-express a variety of embryonic antigens and functions related to their neural-crest pluripotentiality. However, it is possible that all melanocytes respond to these agents, but since no system exists for culturing normal melanocytes this explanation is untestable.

Our results clearly indicate that FSH, melatonin, and NGF affect clonogenic human melanoma cells and suggest that manipulation of these hormones may offer a new avenue to explore in the clinical management of malignant melanoma. We speculate that the heterogeneous clinical nature of melanoma may be related to the expression of responses to trophic hormones related to the neural-crest origin of melanocytes and, thus, that the biological behaviour of melanoma may be explicable (or at least classifiable) in terms of particular trophic hormone responses.

The authors gratefully acknowledge the scientific input of J. Stouffer, B. Magun and J. Trent, the excellent technical performance of L. Berglund, M. Stanfield and D. Sander, and the fine secretarial assistance of R. Collie.

The authors' research is supported in part by Grants CA21839, CA17904, and CA23074 from the National Cancer Institute, National Institute of Health, Bethesda, Maryland.

### REFERENCES

FISHER, R. I., NEIFELD, J. P. & LIPPMAN, M. E. (1976) Oestrogen receptors in human melanoma. *Lancet*, ii, 337.

FULLER, B. B. & MEYSKENS, F. L., JR (1981) Endocrine responsiveness of human melanocytes and melanoma cells in culture. *J. Natl Cancer Inst.* (in press).

GIOVANELLA, B. C., STEHLIN, J. S., SANTAMARIA, C. & 6 others (1976) Human neoplastic and normal cells in tissue culture. I. Cell lines derived from malignant melanoma and normal melanocytes. *J. Natl Cancer Inst.*, **56**, 1131.

HAMBURGER, A. W. & SALMON, S. E. (1977) Primary bioassay of human tumor stem cells. *Science*, **197**, 461.

KITANO, Y. (1976) Stimulation by MSH and dibutyryladenosine 3,5′-cyclic monophosphate of DNA synthesis in human melanocytes *in vitro*. *Arch. Derm. Res.*, **257**, 47.

MEYSKENS, F. L. (1980) Human melanoma colony formation in soft agar. In *Cloning of Human Tumor Stem Cells*. Ed. Salmon. New York: A. Liss. Chapter 8.

MEYSKENS, F. L., BERGLUND, E., FULLER, B., PACELLI, L., SAXE, D. & RAY, G. (1980) Some biological and biochemical properties of a human uveal melanocyte derived line. *In Vitro*, **16**, 775.

MEYSKENS, F. L. & FULLER, B. B. (1980) Characterization of the effects of different retinoids on the growth and differentiation of a human melanoma cell line and selected subclines. *Cancer Res.*, **40**, 2194.

MEYSKENS, F. L. & SALMON, S. E. (1979) Inhibition of human melanoma colony formation by retinoids. *Cancer Res.*, **39**, 4055.

MISHIMA, Y. (1960) Modification of combined DOPA–premelanin reaction. New technique for comprehensive demonstration of melanin, premelanin, and tyrosinase sites. *J. Invest. Dermatol.*, **34**, 355.

PAPANICOLAOU, G. M. (1954) Factors regulating growth and pigmentation of melanoma cells. *J. Invest. Dermatol.*, **66**, 201.

SALMON, S. E. & BUICK, R. N. (1979) Preparation of permanent slides of intact soft agar colony cultures of hematopoetic and tumor stem cells. *Cancer Res.*, **39**, 1133.

SALMON, S. E., HAMBURGER, A. W., SOEHNLEN, B., DURIE, B. G. M., ALBERTS, D. A. & MOON, T. E. (1978) Quantitation of differential sensitivity of human tumor stem cells to anticancer drugs. *N. Eng. J. Med.*, **298**, 1321.

SALMON, S. E., MEYSKENS, F. L., ALBERTS, D. A., SOEHNLEN, B. & YOUNG, L. (1980) New drugs in ovarian cancer and malignant melanoma: *In Vitro* Phase II screening with the human tumor clonogenic cell assay. *Cancer Treat. Rep.* (In press.)

SELBY, P. J., THOMAS, J. M., MONAGHAN, P., SLOANE, J. & PECKHAM, M. J. (1980) Human tumour xenografts established and serially transplanted in mice immunologically deprived by thymectomy, cytosine arabinoside and whole-body irradiation. *Br. J. Cancer*, **41**, 52.

SHERWIN, S. A., SLISKI, A. & TODARO, G. J. (1979) Human melanoma cells have both nerve growth factor and nerve growth factor specific receptors on their cell surfaces. *Proc. Natl Acad. Sci U.S.A.*, **76**, 1288.